# Interventions to enhance the use of Evidence Based Decision Making for Quality Care among Nurses: A Systematic Review

**DOI:** 10.24248/eahrj.v8i1.763

**Published:** 2024-03-28

**Authors:** Safari Agure, Barbara Miyeso, Leyla Abdullahi

**Affiliations:** a Kenya Medical Research Institute (KEMRI), Nairobi, Kenya; b African Institute for Development Policy (AFIDEP), Nairobi, Kenya

## Abstract

**Background:**

Decision-making is the cognitive process that results in the selection of a course of action from several possible alternative options. The complexity of nurses' decisions requires a broad knowledge base and access to reliable sources of information; as well as a supportive working environment therefore requiring that decision making be evidence based with robust knowledge translation platforms to disseminate the evidence. This review aimed to assess interventions for enhancing the use of evidence-based decision making for quality care among nurses.

**Methods:**

This study followed the Preferred Reporting Items for Systematic Review and Meta-Analysis review (PRISMA 2020) checklist. This study protocol was registered with PROSPERO number CRD42021262318.

**Results:**

The search revealed a total of 143 papers divided as follows: PubMed- 65 papers, CINAHL 25 papers and Cochrane 53 papers. In addition, references of included studies were scanned manually for potential papers and another 46 papers extracted. A total of 133 papers were chosen for detailed extraction following removal of 10 duplicate studies.

**Conclusion:**

Results of this review revealed that the interventions that have been used to enhance the use of evidence for decision making are majorly educational. A few interventions have taken the form of modelling, guidelines and programming. Online solutions have also been seen to enhance the use of evidence for clinical practice of nurses.

## BACKGROUND

Decision-making is the cognitive process that results in the selection of a course of action from several possible alternative options. Coming to a choice from a range of options is at the core of decision-making and is key to timely and accurate health service delivery. Decision-making is complex, more so in healthcare; it needs to be context-dependent and is more often characterized by urgency in sometimes less than ideal situations.^1^ Health outcomes are probabilistic rather than certain; as most decisions made are done under conditions of uncertainty.^2^ Hence the importance of these decisions being as right as they can be, remaining critical, as they directly influence health outcomes for patients.

Regrettably, the gap in knowledge about what approaches work best, under what circumstances, and for which patient still remains. More recent developments in health information technology, study methods, and statistical analysis as well as the development of research infrastructure offer opportunities to meet this gap.^3^ Until recently, medical decisions were left in the hands of the physician, however, over the past decades, nurses and patients have been gaining an increasing role in the medical decision making process.^4^ The use of objective facts (Evidence) as the basis for decision-making has recently been seen as a sensible approach. Using evidence in decision-making increases the likelihood of meeting health objectives while revealing inherent risks which can then be mitigated.

It is known that numbers of patients die each year as a result of poor decision-making in healthcare.^5^ This reinforces the importance of quality and timely decision-making in all sectors of Health care.

### Decision Making among Nurses

The complexity of nurses' decisions requires a broad knowledge base and access to reliable sources of information; as well as a supportive working environment therefore requiring that decision making be evidence based with robust knowledge translation platforms to disseminate the evidence.^6^

Clinical decision making done by nurses is notably done in environments filled with uncertainty; making them complex and unpredictable. Decision-making in acute care nursing practice is a complex process. Nurses must consider numerous, potentially competing factors when making decisions to meet patient and family needs.^7^ Hence the requirement to strengthen the component of evidence use to ensure timely, effective and robust decision making among nursing staff to guide clinical practice. Nurses work in larger multidisciplinary teams within the healthcare Clinical decision making done by nurses is notably done in environments filled with uncertainty; making them complex and unpredictable. Decision-making in acute care nursing practice is a complex process. Nurses must consider numerous, potentially competing factors when making decisions to meet patient and family needs.^7^ Hence the requirement to strengthen the component of evidence use to ensure timely, effective and robust decision making among nursing staff to guide clinical practice. Nurses work in larger multidisciplinary teams within the healthcare system, playing a pivotal role in health service delivery and patient outcomes and being in constant contact with the patients. This necessitates them to make judgments and decisions that have direct or indirect impact to patient outcomes; sometimes impacting fatalities.

Research creates knowledge and, in that way, forms an integral part of the knowledge-to-action cycle. However, it is not always the case that research is used as evidence for decision making. For example, in low-resource settings only limited use of local data is done for health-system planning, monitoring, evaluation and decision-making. This is mainly because of limited information sharing and inadequate staff capacity to apply collaborative decision making and analyse and use data for decision-making. Initiated by Florence Nightingale^8^, the nursing profession has more recently provided major leadership for improving care through application of research findings in practice.^9^

### Evidence Based practice

It is important to encourage nurses to actively engage with research evidence during clinical decision making so as to reduce clinical uncertainty. Evidence-based decision making (EBDM) involves prescriptively combining the knowledge arising from one's clinical expertise with patient preferences, and research evidence within the context of available resources.^10^ It involves choosing from a discrete range of options.

In the nursing profession, Evidence based Decision making has come to be termed as Evidence-based practice (EBP). Commensurately, it is defined as the conscientious and judicious use of current best evidence in conjunction with clinical expertise and patient values to guide health care decisions.^11, 12, 13, 14^ The History of EBP among nurses' dates back to the 1800s with Florence Nightingale. Through EBP, nurses can stay updated about new medical protocols for patient care.

By putting into practice evidence learned from research, nurses' care for patients can be made safer.^15^ Nurses need to be proactive in their quest for research knowledge to guide their decisions. This will narrow; possibly close the gap between theory and practice. Utilizing nursing best practice guidelines, reviewing and implementing applicable research evidence, and taking advantage of technological advances are all ways in which nurses can move forward as a well-informed discipline.^16^ In some cases, however, a sufficient research base may not be available. When this is the case, health care decision making is derived principally from non-research evidence sources such as expert opinion and scientific principles.^17^

### Evidence Use Interventions

Various interventions have been applied to enhance Evidence Use in Practice among nurses. The realm of these interventions is broad. They are called Theories, Models, Guidelines, Standards, Training programs, Concepts and or Frameworks. All these interventions however aim to bridge the gap of evidence to action.

### The Theory of Evidence based Medicine (EBM)

The revised and improved definition of evidence-based medicine is “A systematic approach to clinical problem solving which allows the integration of the best available research evidence with clinical expertise and patient values”.^18^ EBM “converts the abstract exercise of reading and appraising literature, into a pragmatic process of using the literature to benefit individual patients while simultaneously expanding the clinician's knowledge base”.^19^ EBM provides a theoretical back drop for EBP.

### Evidence EBP Framework

This framework attempts to answer clinical questions through evaluating the existing evidence.^20^ An offshoot of EBM, EBP is a more specific framework for the nursing profession. EBP is found where clinical expertise, best research evidence and patient values and preferences converge.

EBP is a holistic and patient-oriented approach to health care where a deliberate process of collecting, processing, and implementing research findings for utilization in Clinical Decision Making. EBP aids nurses in identifying strategies that can help solve their patients' problems in the clinical setting, hence encouraging individualized patient care.

### Knowledge Translation

According to The Faculty of Medicine, University of Toronto, Knowledge Translation (KT) is “the effective and timely incorporation of evidence-based information into the practices of health professionals in such a way as to effect optimal health care outcomes and maximize the potential of the health system”. ^21^ The complex theory of KT is now increasingly being used in health-care fields to encompass the process of moving what is learned from research to actual practical circumstances. This process is noted to require continual interaction between knowledge creators and knowledge users.

KT involves having sufficient number of research studies that draw the same conclusion on the same matter before using this information to change practice.^22^

### Cognitive Continuum Theory

The Cognitive Continuum Theory (CCT) is a model of human judgement and decision making aimed at guiding decision-making processes.^23^ This theory has the potential to make major contributions towards understanding the decision-making process of nurses in the clinical environment.^24^ CCT seems to suggest that decision-making is a structured process where decisions are made by assessing the situation and the type of duty or task to be completed, however this is not always the case.

### Guidelines and Standards

Decision making in nursing is guided by a balance of experience, awareness, knowledge, use of assessment tools, influences of colleagues and EBP. The aim of these tools and guidelines is to improve decision making which is critical to quality patient care.

These guidelines are many and include educational guidelines,^25^ Ethical decision-making guidelines,^26^ Guidelines for tools evaluating decision making,^27^ Guidelines for ethical decision making^28^ and Guidelines on evidence-based practice.^29^ Notably, the point of congruence for all these is the emphasis on evidence based decision making as central to nursing services.

As clinical decision-making is a critical component of nursing practice, professional Standards are used to enhance the quality of Nurses' decision making.^30^ “Standards are professionally developed expressions of the range of acceptable variations from a norm or criterion”.^31^ Their main purpose is to guide nurses in the application of their knowledge, skills, and responsibilities in their professional nursing practice.^32^ They are therefore key to developing practice.

### Training Programs

Research in this intervention is not conclusive. A review by Dizon, Grimmer-Somers and Kumar concluded that there was limited evidence relating to the training of allied health professionals in evidence-based practice and learning outcomes.^33^

However, another study by Black et al, concluded that EBP training programs had perceived benefits to the organization. Respondents mentioned the acquired ability to showcase excellence in nursing among the larger healthcare community after undergoing an EBP training program while fostering inter-professional collaboration within their organization, and partnerships between clinicians, administrators and academics.^34^ Similarly, Dizon et al concluded that EBP training programs were effective in enhancing EBP however, noting that changing from current practice to an EBP manner requires more than just basic training.^35^

### How an Intervention Might Work

Various interventions are notably used to strengthen the transfer of research evidence to clinical practices in patient care. Commensurately, these interventions should initiate a shift from intuition-based decision making and increasing evidence-based decision making among nurses.

Examples of hospital-based scenarios that EBP has been seen to work successfully is in infection control, enhancing patient care shift changes, determining nurses' dress codes, in identifying alarm fatigue and in the administration of oxygen therapy.

**FIGURE 1:**
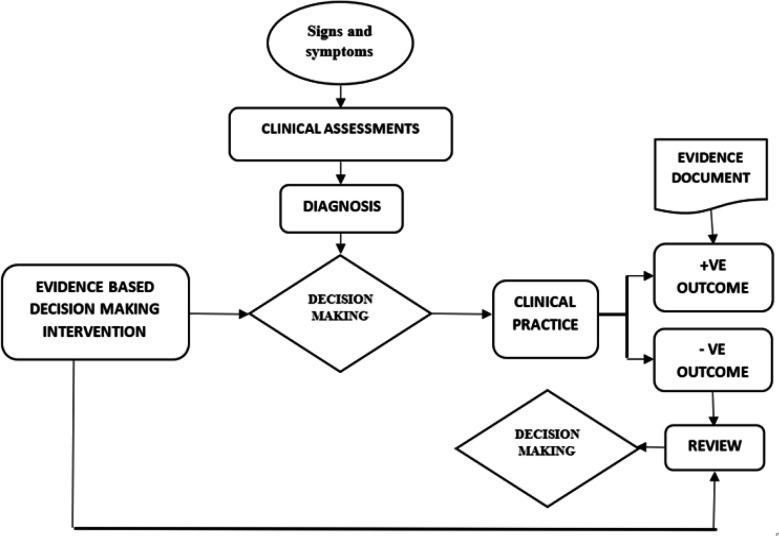
How an Evidence Informed Intervention Would Work

A patient presenting with certain signs and symptoms goes through a series of clinical assessments beginning at triage, a medical doctor's examination and sometimes other specialized tests. These assessments culminate in a diagnosis being made on the point(s) of care that the patient requires. If the patient is to be admitted, the nurses' decision-making processes for clinical practice for that patient begin. For EBP, the nurses would apply their knowledge acquired from current evidence or from past experience while taking into account the patients' views to make these clinical decisions. These decisions most often have a direct impact on patients' outcome whether positive or negative for example management of pain. If the outcome is positive and the patient feels relief from pain, the nurse documents this outcome and the clinical matter is resolved. However, if the outcome is negative, a review of the patients' problem should be carried out, current evidence sought and applied to make a different decision to relieve the pain. This is continuous until the sought relief is attained. See (Figure 1).

### Justification for the Review

Systematic reviews aim to identify, evaluate, and summarize the findings of all relevant individual studies within a given health-related issue. This in turn makes the available evidence more accessible to decision makers. In the area of nursing, systematic reviews are seen to pull together what is already known from research while identifying areas where reviews and more research are necessary, reducing unwanted duplication of research and enhancing clinical practice. Examples of systematic reviews conducted in the field of nurses' decision making have focussed on factors affecting Decision making,^36, 46, 47^ the application of models for clinical judgement,^37^ Shared decision making strategies,^38, 39, 40, 41, 42^ Decision making skills,^43 44^ Measurement of Evidence Informed Decision making skills,^45^ Educational interventions for Clinical decision making^48^ and KT as an intervention for promoting Evidence Informed Decision Making.^49, 50^

The importance of this current review is to get a feel of the types of interventions that have been used to strengthen clinical decision making of nurses. It is noted that there is a myriad of these interventions. However, the congruence or not of these interventions to the best of the authors' knowledge has not been reviewed. There currently exists no study that peruses available literature and analyses these interventions in totality while highlighting their commonality and differences.

### Objective

To assess interventions used to enhance Evidence Informed Decision Making among nurses for patient care.

## METHODS

### Criteria for Considering Studies for this Review

This study followed the Preferred Reporting Items for Systematic Review and Meta-Analysis review (PRISMA 2020) checklist (Table 1).^51^ This study protocol was registered with PROSPERO number CRD42021262318.

**TABLE 1:** Authors' Contributions

Contributions	SA	BM	LA
Research concept and design	√	-	√
Draft the protocol	√	-	√
Develop and run the search strategy	√	-	√
Obtain copies of studies	√	-	-
Select which studies to include	√	√	-
Extract data from studies	√	√	-
Carry out the analysis	√	-	-
Interpret & critic the analysis	√	-	√
Draft the final review	√	-	√

### Types of Studies

Types of studies that were assessed include intervention studies, operation research studies, implementation research studies, descriptive studies and other research reviews.

### Types of Participants

This review considered studies involving nurses both registered nurses (RNs) and nursing practitioners (NPs) as well as student nurses who delivered primary health care to patients.

### Types of interventions

Guidelines and Standards; Training Programs; Models and theories; Frame works

### Types of outcome measures

#### Primary outcomes

- Knowledge, attitudes and perceptions of Nurses on Interventions for enhanced Clinical Decision making

- Enhanced decision making for clinical practice among nurses

- Cost of the intervention and how it impacts its access by nurses

- Proportion of nurses who benefit from these interventions

#### Secondary outcomes

- Quality of care.

### Search Strategy

A systematic search of literature published on the use of evidence to guide decision making and clinical practice among nurses was performed. Databases that were searched for papers reporting primary studies in the area were PubMed, CINAHL, and Cochrane. The search and extraction were done between September 2021 and Dec 2021 with no time and language limit.

MESH terms used during the search included nurses, evidence-based decision making, outcomes such as quality of care, intervention or their synonyms (Nurse Practitioners (NP), Nurse Consultants, Clinical Nurse Specialists, Certified specialist nurse, Registered nurse (RN), family nurse, Critical care nurse; Evidence informed decision making, EBP, Decision making; Quality Care, Standard of care, Patient satisfaction and Models, Frameworks, Protocols, SOPs, Manuals, Guidelines, Theories respectively. Boolean search terms included AND to include only relevant results that contain the required keywords: Decision making, Nurses, Clinical practice, OR to include both Evidence based, Evidence informed and other term synonyms (Table 2).

**TABLE 2:** Search Terms Used

Key terms	Search strategy
Nurses	Nurse Practitioners (NP), Nurse Consultants, Clinical Nurse Specialists, Certified specialist nurse, Registered nurse (RN), family nurse, Critical care nurse
Outcomes	Quality Care, Standard of care, Patient satisfaction
Evidence based decision making	Evidence informed decision making, Evidence Based Practice, Decision making
Intervention	Models, Frameworks, Protocols, SOPs, Manuals, Guidelines, Theories

In addition, we searched for primary studies that included interventions for enhancing evidenced based or informed decision making and or practices by nurses. Any initial discrepancies between the screening authors (SA and BM) were resolved by discussions and consensus building. A third reviewer (LA) was available in the event of consensus not being reached, but this was not required as there emerged no major disagreement.

### Selection of Studies

**FIGURE 2:**
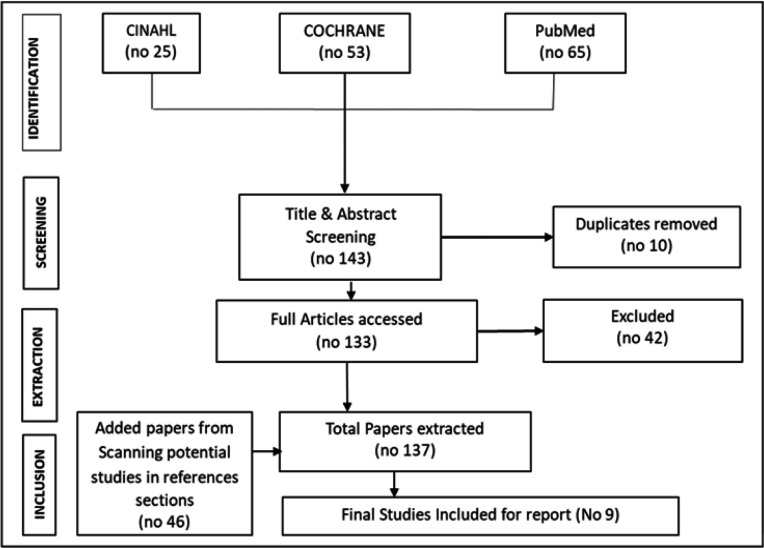
PRISMA Flow Chart

We conducted a 2-step screening process between two authors (SA and BM). Firstly, titles and abstracts was screened for eligible studies. Thereafter, a full text of eligible studies was obtained for further review and final selection of studies to include and those to exclude (Tables 3 & 4). We resolved any disagreements regarding the inclusion of studies by discussion or consultation of a third review author (LA). We used the PRISMA flow chart to summarise the search and selection of studies for the review (Figure 2).

**TABLE 3:** Included Studies

	Author (s)	Country	Cadre of nurses	Intervention	Outcome measures	Study design	Major conclusions
1	Gigli KH et al 2020	United States	ICU nurses	Education & Certification	Improved patients care	Cross sectional survey	Nurses working in the intensive care unit & having a specialty certification was associated with their individual psychosocial beliefs & perceptions of evidence-based practices, however education level was not.
2	Samuel & Fetzer et al 2009	United States	Clinical nurses	Samuels' Pain Management Documentation Rating Scale	Evidence-based pain management	Descriptive retrospective	Expertise may impact the implementation of evidence especially in areas where practice patterns are well established.
3	Laibhen Parkes 2014	United States	BSN-prepared nurses	Web-based EBP educational module	Competency in Evidence based practice	Mixed methods	Creative strategies that promote a culture of clinical inquiry & motivate nurses to use the EBP resources available are warranted. Ultimately, improving EBP competence in this population will increase the capacity of paediatric nurses in achieving the IO M's 2020 goal of basing 90% of clinical decisions made on the best evidence.
4	Petursdottir et al 2019	Iceland	Palliative care nurses	Educational program	Quality of care	Mixed Methods	An advanced educational intervention programme was successful in improving the nurses' knowledge, skills, satisfaction & confidence in relation to applied family nursing approach within the context of caring for families affected by advanced/final stage cancer.
5	Holland, Knafi & Bowles, 2013	Not clear	General nurses	evidence-based discharge planning (DP) decision support	Discharge planning	Cross Sectional	to describe the ability of an evidence-based discharge planning decision support tool to identify and prioritize patients appropriate for early discharge planning intervention. Specifically, we aimed to determine whether patients with a high ESDP score report more problems and continuing care needs in the first few weeks after discharge than patients with low ESDP scores.
6	Edwards et al 2017	Kenya, Uganda, South Africa, Jamaica	HIV nurses	Clinical practice Guidelines	Standard of care	Qualitative	Guidelines should more consistently acknowledge diverse implementation contexts, propose how recommendations can be adapted to these realities, and suggest what role frontline healthcare providers have in realizing the structural changes necessary for healthier work environments & better patient care. Guideline recommendations should include more explicit advice on adapting their recommendations to different care conditions.
7	Branham et al 2014	United States	Critical care	evidenced based	EBP use	Qualitative	nurses practice course there was no difference in evidence-based practice use in ACNBs who had a dedicated evidenced based practice course when compared to those who had it integrated throughout the curriculum. Regardless of instructional methodology, it is important for all health care providers to have formal training in the use of EBP. Ultimately, the goal of ACNP practice is to attain the best clinical outcome for the individual patient and family.
8	Wallen et al 2010	United States	General nurses	Mentorship program	EBP skills	Mixed Methods	An EBP Mentorship Programme comprised of a series of intensive workshops with ongoing EBP skills building activities can have positive effects on nurses' perceptions of organizational culture, their EBP beliefs and implementation, as well as job satisfaction and intent to leave their profession.
9	Munroe et al 2008	United States	General nurses	Web based	Behaviour change	Mixed methods	having a mentor leads to stronger beliefs and greater implementation by nurses as well as greater group cohesion, which is a potent predictor of nursing education turnover rates.

**TABLE 4:** Excluded Studies

Study ID	Reason for exclusion
Clarke et al 2021	The study is a review
Ryder et al 2020	The study is a review and does not have an intervention
Sayılan 2019	The study is a review
MaryBeth et al 2011	The study is a review
Bourgault et al 2018	This is an article
Study did not have any intervention	A Student thesis
Salinas et al 2019	Was unable to get the full paper
Roberge et al 2016	Participants include other health workers beyond nurses
Mackie et al 2018	No mention of Evidence based decision making or EBP
Ryan EJ 2016	Is a review
LaSala et al 2007	Only abstract was available
Trochelman et al 2012	Participants were patients
Darmody J.V. 2008	Only abstract was available
Taylor, Suzanne 2015	A Student thesis
Kaplan & Frosch 2005	Participants were doctors
Peters et al, 1999	Participants were members of the public
Wilson et al 2005	No mention of Evidence based decision making or EBP
Julie Gassaway 2010	No mention of Evidence based decision making or EBP
Redi et al, 2011	A Student thesis
Wang, Chien & Lee 2012	Only abstract was available
no authors	A conference paper
Shing et al 2015	multiple respondents
Gold et al 2015	multiple respondents
Klafke et al 2016	Only abstract was available
Thompson 2004	Is an article
Klimm et al 2008	Participants were general practitioners
Towler J 2001	Is an article
Gridelli et al 2004	Is a meeting report
Dagenais et al 2008	Only abstract was available
Blair et al 2017	Only abstract was available
Kew et al 2017	Study was a review
He HG et al, 2014	No mention of Evidence based decision making or EBP
Isselhard et al 2020	Incomplete study
Légaré et al 2018	Study was a review
Giguere etc al 2018	multiple respondents
Daniel Cardoso 2018	Not completed RCT
MacDermid et al 2012	multiple respondents
Butler KD 2011	Only abstract was available
Ciliska et al 2001	Only abstract was available
Cruz et al 2016	Study did not have any intervention
Fridman & Frederickson 2014	Only abstract was available
Irwin et al 2013	Only abstract was available
Melnyk et al 2004	Study did not have any intervention
Gerrish et al 2012	Study did not have any intervention
Gunes 2017	Paper not available
Titler et al 2001	This was an article
Rosswurum & Larrabee 1999	Study was a review

### Paper Selection and Extraction

A data extraction form was adapted and used by the research team. The data extracted included the following key descriptions: setting of the study (country); type of study; type of participants (cadre of nurse); type of intervention (training programs, models and theories, guidelines and standards, frame works), type of outcomes measured (quality of care, standard of care), key study aims and conclusions, ethical considerations, gender of respondents and the duration of the study.

Two reviewers (SA and BM) independently used the CASP tools to determine whether the research papers included met the criteria of being free of bias and relevant to the matter at hand.^52^

### Assessment of Risk of Bias in Included Studies

Each of the included studies were assessed for risk of bias using the CASP (Critical Appraisal Skills Program) risk of bias tool according to the type of study. CASP has appraisal checklists designed for use with Cohort Studies,^53^ Case Control Studies^54^ and Qualitative studies.^55^ The other appraisal tools used were Cross sectional study tool^56^ and mixed method risk assessment tool^57^ (Tables 5, 6, 7).

**TABLE 5:** Risk of Bias Assessments for Qualitative Studies

Study ID	Is there an adequate rationale for using a mixed methods design to address the research question?	Are the different components of the study effectively integrated to answer the research question?	Are the outputs of the integration of qualitative & quantitative components adequately interpreted?	Are divergences & inconsistencies between quantitative & qualitative results adequately addressed?	Do the different components of the study adhere to the quality criteria of each tradition of methods involved?	Overall risk of bias
Laibhen Parkes 2014	Low risk	Low risk	Low risk	Not clear	Low risk	Low risk
Petursdottir et al 2019	Low risk	Low risk	Low risk	Low risk	Low risk	Low risk
Wallen et al 2010	Low risk	Not clear	Low risk	Not clear	Low risk	Moderate risk
Munroe et al 2008	Low risk	Low risk	Low risk	Not clear	Low risk	Low risk

**TABLE 6:** Risk of Bias Assessment for Cross Sectional Studies

Study ID	Were the aims clear?	Was the study design appropriate?	Was the sample size justified?	Was the target/reference population clearly defined?	Was the sample frame taken from an appropriate population	Was the selection process representative of the target population?	Were measures undertaken to address non-responders?	Were the risk factor and outcome variables measured appropriately t?	Were the risk factor and outcome variables measured correctly?	Is it clear what was used to determined statistical significance estimates?	Were the methods (including statistical methods) sufficiently described?	Were the basic data adequately described?	Does the response rate raise concerns about non-response bias?	If appropriate, was information about non-responders described?	Were the results presented for all the analyses described in the methods?	Were the authors' discussions and conclusions justified by the results?	Were the limitations of the study discussed?	Were there any funding sources or conflicts of interest that may affect' interpretation?	Was ethical approval or consent of participants attained?	Overall risk of bias
Gigli KH et al	Low risk	Low risk	Not clear	Low risk	Not clear	Not clear	Low risk	Low risk	Low risk	Low risk	Low risk	Low risk	Not clear	High risk	Low risk	Low risk	Low risk	High risk	Low risk	Low risk
Samuel & Fetzer et al 2009	Not clear	Low risk	High risk	Low risk	Not clear	Not clear	Not clear	Low risk	Not clear	High risk	Low risk	High risk	Not clear	Not clear	Not clear	Not clear	High risk	Not clear	Not clear	High risk
Holland, Knafi & Bowles, 2013	Low risk	Low risk	Low risk	Low risk	Low risk	Not clear	High risk	Not clear	Not clear	Low risk	Low risk	Low risk	Low risk	Low risk	Low risk	Low risk	Low risk	High risk	Low risk	Low risk

**TABLE 7:** Risk of Bias Assessment for Qualitative Studies

Study ID	Was there a clear statement of the aims of the research?	Is a qualitative methodology appropriate?	Was the research design appropriate to address the aims of the research?	Was the recruitment strategy appropriate to the aims of the research?	Was the data collected in a way that addressed the research issue?	Has the relationship between researcher and participants been adequately onsidered?	Have ethical issues been taken into consideration?	Was the data analysis sufficiently rigorous?	Is there a clear statement of findings?	How valuable is the research?	Overall risk of bias
Edwards et al 2017	low risk	low risk	low risk	unclear	low risk	unclear	low risk	low risk	low risk	low risk	Low risk
Branham et al 2014	low risk	low risk	low risk	low risk	low risk	low risk	low risk	low risk	low risk	low risk	Low risk

### Data Synthesis

We pooled data from studies of similar study designs, similar interventions, similar participants, and similar outcomes in a meta-analysis using the random-effects model if there was no significant statistical heterogeneity, methodological difference, or high risk of bias. For outcomes with substantial variation between studies in the reported interventions, participants, study designs, and outcome measures, we did not pool the results but summarised the findings in a narrative format. Overall, we interpreted the study findings by taking into account the methodological quality of the studies and the strength of the evidence. For each observed effect, we explicitly stated the strength of evidence and drew conclusions.

### Subgroup Analysis, Sensitivity Analysis and Investigation of Heterogeneity

Sub-group analyses were not conducted as the data was not sufficient. In addition, we did not conduct sensitivity analysis as the data was not sufficient.

## RESULTS

### Paper Selection and Extraction

The search revealed a total of 143 papers divided as follows: PubMed- 65 papers, CINAHL 25 papers and Cochrane 53 papers. In addition, references of included studies were scanned manually for potential papers and another 46 papers extracted. A total of 133 papers were chosen for detailed extraction following removal of 10 duplicate studies. Full texts of these selected articles were examined, details of and data extracted in duplicates using a developed data extraction form. Original articles that reported nurses' use of an evidence intervention in English were included. Papers on reviews, any secondary analysis and articles were excluded. Also excluded were papers which did not have nurses as exclusive respondents and did not involve the use of an intervention to enhance evidence use (Figure 2).

### Characteristics of Included Papers

**Population:** The population in this study were all cadres of nurses and included oncology nurses, paediatric nurses, general nurses, nurse practitioners as well as student nurses.

**Intervention:** Included studies, revealed that the evidence enhancing interventions that have been used by nurses include educational enhancing interventions.^58, 60, 64, 59^ Scales and models,^61^ Guidelines,^63^ Online solutions,^60, 65^ evidence-based discharge planning (DP) decision support^66^ and a Mentorship program.^67^ These interventions span several domains including, palliative care, critical care, paediatric care, infection care, patient discharge.

**Comparator:** There were no comparators in this study.

**Outcome:** Outcomes measured by the studies included quality of care,^58, 64^ improved patient care,^58^ standards of care^63^ and Evidence based practices like patient discharge, skills and competencies.^66, 59, 60, 61, 67, 65^

**Study designs:** Majority of the studies were conducted as Mixed methods studies; These were.^65, 64, 60, 67^ Others employed Cross sectional designs^58, 66^ and others used Qualitative designs.^59, 63^ Only one study utilized a Descriptive design methodology.^61^

### Effect of Interventions

### Quality of Care and Improved Patient Care

In the area of Quality of care and Improved patient care, Gigli KH et al 2020 highlighted the use of Education and certification as interventions that can influence evidence-based practices in the intensive care unit (ICU). Certification was determined as more effective that education in improving self-efficacy and certain clinical practices. As such a conclusion to the study was given as, ‘supporting nurses in obtaining specialty certification could assist with the adoption of evidence-based practices as a means to improve quality of care in the intensive care unit.^58^

Petursdottir studied the use of an educational program to enhance evidence use and influence quality of care under palliative care settings. Results of the study realized a significant increase in the critical appraisal skills of the nurses in their clinical practices after their being a part of the educational program. Also improved were the nurses' knowledge of, skills in, satisfaction and confidence in EBP.^64^

Using a quasi-experimental mixed methods study Wallen et al, used a mentorship program to influence evidence based clinical practices. The researchers noted that nurses' beliefs influenced EBP practices. Analysis of the intervention effects showed that nurses who were given mentors have stronger beliefs in EBP that led them to pursue evidence in their practices.^67^

### Standard of Care

The use of evidence based interventions to determine standards of care was demonstrated by Edwards et al 2017. While focussing in the area of HIV workplace safety in Kenya, Uganda, Jamaica and South Africa, it was noted that in order to strengthen the potential of guidelines to influence practice they need to be more explicit, encompass diverse implementation scenarios while proposing how to adapt to them and elucidate the role of frontline health workers in implementing them for improved standards of care.^63^

### Best Practices

Several best practices were identified as guided by evidence including EBP, best practices in clinical pain management, EBP use and competencies, patient discharge systems and engagement with research discussion forums.

Braham et al studied the use of EBP as an intervention in clinical care. The study participants noted that EBP was the best way to deliver care. However, caution was given in that ‘real world practice' would require improved instruments and educational strategies for integration of EBP and Acute care nursing.^59^

While focusing on evidence based pain management especially documentation of the same as a best practice, Samuel & Fetzer et al 2009 studied the impact of documentation in a Pain Management scale as an intervention to influence practices of completion of the pain documents and patterns. Clinical expertise was concluded as having impacts on completion of pain management documentation and recommending that implementation strategies for implication needs to target different expertise levels differently.^61^

Laibhen Parkes 2014 sought to assess and refine a web based EBP educational interventions that can improve competency in Evidence based practices in the area of paediatrics. According to the authors, individual beliefs can influence competencies. The study revealed that Web Based educational interventions for EBP are moderately feasible, acceptable and applicable.^60^

Educational interventions for their impact on skills and competencies for EBP were studied by Munroe et al. The study conducted among nurse managers, revealed that while the nurses had a positive attitude towards EBP, educational interventions used were not significantly impactful on attitudes, knowledge and skills for practice.^65^

Diane E Holland, George J Knafl and Kathryn H Bowles conducted a study that applied an evidence-based discharge planning tool as an intervention to support decision making for when prioritizing patients for early discharge. This tool was found to be effective in reducing bias, while promoting efficiency. This has the potential to contribute immensely toward continuum of care for patients.^66^

### Risk of Bias Assessments for Included Studies

We had 9 observational studies (4 mixed studies, 3 cross-sectional studies and 2 qualitative studies). The summary of the various risk of bias assessments are detailed in Table 5.

## DISCUSSIONS

Evidence based practices for clinical care have been well embraced in nursing practices. According to Verloo et al, the implementation of EBP in daily health care practice is strongly encouraged as it is widely recognized as a means to improve the quality and safety of health care for patients and reduce avoidable costs.^68^ The use of evidence in healthcare began with Evidence based medicine and now among nurses, Evidence based decision making has become synonymous with EBP.

Following that, the aim of this study which was to identify and describe the interventions that have been used by nurses to enhance their use of evidence in clinical practice was fulfilled through the systematic review of primary research in the area. This paper sought to highlight the various interventions that are being used to enhance the use of evidence to guide clinical care in various medical areas. Results of this review revealed that the majority of interventions used to enhance evidence based decision making, are predominantly educational or a form of teaching module, a guide/guideline or a summary model of steps to take. Our findings indicate that most of the interventions that have been used to enhance evidence use in decision making and clinical practice have leaned towards education and capacity building.

This study has revealed that educational programs through training and mentorship are the most utilized interventions to enhance EBDM and EBP among nursing staff. These have been seen as impacting behaviour change and attitudes towards acquisition and use of evidence and in turn impacting patient outcomes positively. Educational interventions work towards imparting knowledge and skills necessary for EBP while impacting practice and changing attitudes and behaviour of nurses.^65^ So important is this issue that authors in this area have called for the integration of teaching EBP skills in the nursing curricula.^64, 67, 59^ Notably, these interventions can be delivered using electronic means via the web or alerts.^60^

Guidelines used to enhance EBP are systematically developed statements to assist practitioner and patient decisions about appropriate health care for specific clinical circumstances.^63^ Guidelines are seen to provide a summary of the relevant medical literature and offer assistance in deciding which diagnostic tests to order, which treatments to use for specific conditions, when to discharge patients from the hospital, and many other aspects of clinical practice.^66^

## CONCLUSIONS

Results of this review revealed that the interventions that have been used to enhance the use of evidence for decision making are majorly educational. A few interventions have taken the form of modelling, guidelines and programming. Online solutions have also been seen to enhance the use of evidence for clinical practice of nurses. This approach helps ensure that nursing practices are based on sound evidence and research, ultimately leading to better patient outcomes.

The review successfully identified and assessed studies related to interventions aimed at improving Evidence-Based Decision making among nurses, with a focus on patient care. It highlighted that the nursing field widely embraces the use of evidence to inform practice. This is noted to be particularly the case in the US, pointing to the need for more research in this area in Low- and Middle-Income Countries so as to enhance evidence based practise in such countries. This review only found one paper on research conducted in Africa on evidence use by nurses. Tailoring interventions and their usage in this part of the world will require implementation and impact research.

As it is clear that evidence should guide practice and will lead to improved patient outcomes. In terms of strengthening service delivery at the clinical level, evidence plays a big role and evidence based best practices need to be adopted far and wide.

### Study Limitations

Very few primary studies have been conducted in the area of EBP interventions which is reflected in the small number of studies included. We cannot therefore assume that the search strategy found all potentially eligible studies. That many of the few identified studies had paid access to full articles hampered the inclusion of some eligible papers. The authors did not contact article/paper authors for access where only abstracts were available. Only papers which were open access were accessed.
